# Treatment of pectus carinatum with a brace – a case report

**DOI:** 10.1186/1748-7161-7-S1-P18

**Published:** 2012-01-27

**Authors:** HR Weiss, M Werkmann

**Affiliations:** 1 Orthopedic Rehabilitation Service, Gensingen, Germany

## Background

Measurement of thoracic deformities is difficult and therefore little information is available about the effectiveness of conservative treatment. While from a specialized center in Brasil positive case reports have been released for the treatment of all kinds of genuine thoracic deformities with the help of braces [1], the German health insurances in part do reject the costs of such treatment due to the lack of scientific studies. Aim of this case report was to demonstrate the effectiveness also of the (smaller) German standard of braces for the treatment of genuine thoracic deformities.

## Case report

A 15 year old boy with pectus carinatum agreed to be braced with a custom made pectus carinatum brace. Pictures were made at the start of treatment, at some follow-up presentations and at weaning at the age of 17. The documentation clearly shows nearly full recovery, which means the deformity is hardly visible. It also has been shown that most of the result was gained during the first 6 months of treatment.

## Conclusions

1. A case series of patients treated with pectus carinatum braces seems worthwhile.

2. Brace treatment should be performed during times of accelerated growth.

3. Brace treatment of patients with thoracic deformities is indicated to warrant the patients social participation.
